# Genetic tools for the stable overexpression of circular RNAs

**DOI:** 10.1080/15476286.2022.2043041

**Published:** 2022-03-15

**Authors:** Nicol Mecozzi, Arianna Nenci, Olga Vera, Ilah Bok, Aimee Falzone, Gina M. DeNicola, Florian A. Karreth

**Affiliations:** a Department of Molecular Oncology, H. Lee Moffitt Cancer Center and Research Institute, Tampa, FL, USA; b Cancer Biology PhD Program, University of South Florida, Tampa, FL, USA; c Gene Targeting Core Facility, H. Lee Moffitt Cancer Center and Research Institute, Tampa, FL, USA; d Department of Cancer Physiology, H. Lee Moffitt Cancer Center and Research Institute, Tampa, FL, USA

**Keywords:** Circular RNA, transposon, melanoma, mouse model, ESC-GEMM, sleeping beauty, piggyBac

## Abstract

Circular RNAs (circRNAs) are a class of non-coding RNAs featuring a covalently closed ring structure formed through backsplicing. circRNAs are broadly expressed and contribute to biological processes through a variety of functions. Standard gain-of-function and loss-of-function approaches to study gene functions have significant limitations when studying circRNAs. Overexpression studies in particular suffer from the lack of efficient genetic tools. While mammalian expression plasmids enable transient circRNA overexpression in cultured cells, most cell biological studies require long-term ectopic expression. Here we report the development and characterization of genetic tools enabling stable circRNA overexpression *in vitro* and *in vivo*. We demonstrated that circRNA expression constructs can be delivered to cultured cells via transposons, whereas lentiviral vectors have limited utility for the delivery of circRNA constructs due to viral RNA splicing in virus-producing cells. We further demonstrated ectopic circRNA expression in a hepatocellular carcinoma mouse model upon circRNA transposon delivery via hydrodynamic tail vein injection. Furthermore, we generated genetically engineered mice harbouring circRNA expression constructs. We demonstrated that this approach enables constitutive, global circRNA overexpression as well as inducible circRNA expression directed specifically to melanocytes in a melanoma mouse model. These tools expand the genetic toolkit available for the functional characterization of circRNAs.

## Introduction

The last decade of RNA research revealed a myriad of non-coding RNAs that support fundamental biological processes and whose deregulation may contribute to the development of diseases such as cancer. Circular RNAs (circRNAs) are among the most recent additions to the non-coding RNA compendium and represent a peculiar class of RNA molecules. Generated from pre-mRNA via a process termed backsplicing where the 3’ end of a downstream exon is spliced to the 5’ end of an upstream exon, circRNAs form covalently closed ring molecules lacking 5’ CAP and 3’ polyA tail features. The circular structure protects also from exonuclease cleavage, rendering circRNAs extraordinarily stable. Advances in RNA sequencing led to the identification of thousands of circRNAs in humans and other species. Interestingly, circRNA expression is cell type- or differentiation state-dependent and often uncoupled from the expression of their cognate linear RNAs from which they derive [1–10]. This suggests that circRNA formation is a regulated process and that circRNAs must serve important functions in the cell. Indeed, circRNAs have been shown to act as natural microRNA sponges [3,11], associate with RNA-binding proteins [12–16] and chromatin [17], and regulate the transcription of their parental genes [18–20]. Some circRNAs even contain internal ribosome entry site-like structures or N6-methyladenosine modifications to recruit the translation machinery and encode proteins [21–27]. Thus, circRNA functions are diverse and unpredictable based on their sequence alone, requiring functional characterization to reveal the underlying mode of action.

Functional analyses of circRNAs have been hindered by technical challenges. Knock-down experiments using RNAi require that the siRNA or shRNA is targeted to the backsplice junction to avoid simultaneous silencing of the cognate linear mRNA. This limits the possibilities for designing potent siRNA/shRNA and may lead to off-target effects on the cognate linear mRNA or other unrelated transcripts. Recently, a CRISPR approach using an engineered type VI-D Cas13d enzyme, CasRx, was developed to silence RNA transcripts [28]. Using an extended guide RNA placed over the backsplice junction, this CRISPR approach demonstrated superior circRNA silencing in terms of efficiency and specificity [29]. The overexpression of a circRNA of interest may be even more difficult than silencing. Circularization requires the presence of flanking introns containing repeat elements, which upon their alignment bring the appropriate splice sites into close proximity to aid in the backsplicing reaction. Mammalian expression plasmids have been generated where circRNA exons are flanked by introns derived from genes that efficiently produce circRNAs, for instance the *D. melanogaster* Laccase2 introns or the human ZKSCAN1 introns [30,31]. With these expression plasmids efficient ectopic circRNA expression can be achieved in mammalian or *D. melanogaster* cells [30]. However, such plasmids do not integrate into the genome and ectopic circRNA expression is therefore only temporary. This significantly limits long-term phenotypic analyses of cell biological effects. In addition, whether similar approaches are amenable to circRNA overexpression in genetically engineered mice for functional circRNA studies *in vivo* is not known.

Here, we tested stable, long-term circRNA overexpression approaches. While lentiviral circRNA delivery performed poorly, we found that using a transposon for delivery and genomic integration of circRNA expression constructs enables long-term circRNA expression in cells and a hepatocellular carcinoma mouse model. Moreover, we generated genetically engineered mice that overexpress a circRNA either constitutively and ubiquitously or inducibly in a cell-type specific manner in a melanoma model. These tools and approaches will enable more in-depth analyses of circRNA functions *in vitro* and *in vivo*.

## Materials and methods

### Plasmids and cloning

To generate spGFP-pLenti, pcDNA3-ZKSCAN1-spGFP (Addgene) was digested with BamHI (New England Biolabs, Cat. # R3136) and XhoI (New England Biolabs, Cat. # R0146) and the spGFP-ZKSCAN1 cassette ligated into BamHI/SalI-digested pLenti-EF1α-GFP_miRE to replace GFP_miRE (SalI, New England Biolabs, Cat. # R3138). The EF1α-spGFP-ZKSCAN1-Blast transposon was generated by digesting the ATP1 transposon (provided by Roland Rad) with BstXI (New England Biolabs, Cat. # R0113) to remove the CAGS promoter, polyadenylation signals, and splice acceptor/donor sites and replace them with a multiple cloning site containing MluI-NotI-BglII sites by oligo cloning (New England Biolabs, Cat. #s R3198, R3189, R0144 respectively). A SV40 polyA-PGK promoter-BlasticidinR cDNA-bGH polyA cassette was then synthesized by GeneArt (Thermo Fisher), PCR amplified, and InFusion (Clonetech/Takara, Cat. # 638911) cloned into NotI/BglII digested ATP1-MCS, generating ATP1-Blast. Finally, EF1α-spGFP-ZKSCAN1 was PCR amplified from spGFP-pLenti and InFusion cloned into MluI/NotI digested ATP1-Blast. To generate the EF1α-spGFP targeting vector, a EF1α-LSL-GFP targeting vector was digested with XhoI/EcoRI (EcoRI, New England Biolabs, Cat. # R3101). The spGFP-ZKSCAN1 cassette was PCR amplified from spGFP-pLenti to introduce restriction sites, the PCR product was digested with XhoI/EcoRI, and ligated into the targeting vector. To generate the TRE-spGFP targeting vector, the spGFP-ZKSCAN1 cassette was PCR amplified and InFusion cloned into XhoI/EcoRI digested cTRE-CHC targeting vector (provided by Lukas Dow). The circPMS1 and circDNAJC2 transposons were cloned by synthesizing the circRNA exons by GeneArt, followed by PCR amplification and InFusion cloning into SnaBI/PstI (New England Biolabs, Cat. #s R0130 and R3140, respectively) digested EF1α-spGFP-ZKSCAN1-Blast transposon plasmid.

### Cell culture

Human melanoma cell lines were cultured in RPMI-1640 (Lonza, Cat. # BW12-702 F) containing 5% FBS (VWR, Cat # 76236–284), NIH3T3 in high-glucose DMEM (VWR, Cat. # 45000–304) containing 10% bovine calf serum (VWR, Cat # 16777–206), and HEK293T-LentiX in high-glucose DMEM (VWR, Cat. # 45000–304) containing 10% FBS in a 5% CO_2_ incubator at 37°C. For Crystal Violet staining (VWR, Cat. # 97061–850), cells were washed in phosphate-buffered saline (PBS) (VWR, Cat # 95042–492) and stained with 0.1% Crystal Violet in 20% methanol. The dye was extracted with 100 µL of 10% acetic acid and absorbance was measured at 600 nm.

### Transfection and virus production

For transposon transfection, 0.9 µg of spGFP transposon and 0.1 µg of either pCMV-HSB5 (Sleeping Beauty transposase), pCDNA-mPB (piggyBac transposase), or empty pCDNA3.1 were transfected into HEK293T-LentiX or melanoma cells at 80–90% confluence using JetPrime (Polyplus, Cat. # 89129–924) according to the manufacturer’s recommendations. pCMV-HSB5 and pCDNA-mPB were provided by David Adams. Where indicated, transfected cells were selected with 10 µg/mL Blasticidin (Invivogen, Cat # ant-bl-1) for 10 days starting 24 hours following the transfection. For lentivirus production, HEK293T-LentiX cells were transfected at 90% confluence with 6 µg of lentiviral vector, 0.66 µg of VSVG envelope vector, and 5.33 µg of Δ8.2 packaging vector using JetPrime according to the manufacturer’s recommendations. Viral supernatant was collected after 48 hours, filtered through 0.45 µm syringe filters (VWR, Cat # 28145–505), and used to transduce melanoma cells in the presence of 8 µg/mL polybrene (Sigma-Aldrich, Cat # 107689). Viral titre was determined using the NucleoSpin RNA Virus (Takara, Cat. # 740956.10) for RNA isolation and Lenti-X RT-qPCR Titration Kits (Takara, Cat. # 631235) for titre measurement following the manufacturer’s recommendations

### RNA isolation, cDNA synthesis, and quantitative real-time PCR

For RNA isolation from cell lines, cells were washed with PBS and collected by scraping and centrifugation. Cell pellets were resuspended in TRI reagent (Zymo Research, Cat. # R2050-1-200) and RNA was isolated as per the manufacturer’s instructions. For RNA isolation from organs and tumours, tissue pieces were physically disrupted in 600 µL of TRI reagent in high-impact zirconium beads (Benchmark, Cat. # D1032-30) using a microtube homogenizer (BeadBug, Cat. # SKU:BS-BEBU-3). RNA was then isolated from lysate diluted 1:2–1:3 in TRI reagent according to the manufacturer’s recommendations. RNA was treated with RQ1 RNase-free DNase (Promega, Cat. # M6101) following the manufacturer’s instructions. cDNA was synthesized using the 5X PrimeScript RT Master Mix (Clontech/Takara, Cat. # RR036A), following the manufacturer’s instructions. RT-qPCRs were performed using PerfeCTa SYBR Green FastMix (Quantabio, Cat. # 101414–278) and results were analysed using the ΔCt method and Actin was used as a normalization control. The following primers were used: spGFP forward 5’-ATGGCAACATCCTGGGCAAT-3’, reverse 5’-TTCACATCGCCATTCAGCTC-3’; circPMS1 forward 5’-TCCAAGATCTCCTCATGAGC-3’, reverse 5’-TACAACACTGACCACCGAAG-3’; circDNAJC2 forward 5’-AGAAGCTGCTCGGTTAGCTA-3’, reverse 5’-GTGCTCTTGTTGCTCTGTTC-3’; Actin forward 5’-TTGCTGACAGGATGCAGAAG-3’, reverse 5’-ACATCTGCTGGAAGGTGGAC-3’.

### Western blot

Cells were lysed in RIPA buffer and 20 μg of protein lysate were separated on NuPAGE 4–12% Bis-Tris Midi Gels (Life Technologies, Cat. # WG1402BOX). Proteins were transferred onto nitrocellulose membrane and stained with Ponceau Red to confirm complete transfer. Membranes were blocked in 5% non-fat dry milk in TBS-T and incubated over night at 4°C with anti-GFP (1:1,000; Cell Signalling Technologies, Cat. # 2956) or anti-Actin (1:4,000; Invitrogen, Cat. # AM4302) antibodies diluted in 5% non-fat dry milk in TBS-T. The membranes were washed with TBS-T and incubated with HRP-conjugated secondary anti-rabbit antibody (1:20,000; Jackson ImmunoResearch, Cat. # 115-035-003) for 1 hour. The signal was detected with SuperSignal West Pico PLUS Chemoluminescent Substrate (Thermo Scientific, Cat. #34577) following the manufacturer’s instructions.

### Southern blot

A375 cells were transfected with 0.9 µg of spGFP transposon and 0.1 µg of pCDNA-mPB (piggyBac transposase) and selected with 10 µg/mL Blasticidin for 3 weeks. Clones derived from single cells were picked using cloning cylinders and expanded. Genomic DNA was isolated with Proteinase K lysis buffer and 15 µg of DNA were digested overnight with 40 units of MspI (New England Biolabs, Cat. # R0106). Digested DNA was separated on a 0.8% agarose gel, followed by in-gel depurination, denaturation, and neutralization. DNA was capillary transferred in 10xSSC onto nitrocellulose membrane and crosslinked by baking at 70°C for one hour. A Blasticidin probe was PCR amplified, labelled with α-^32^P-dCTP using DECAprime II random prime labelling kit (Invitrogen, Cat. # AM1456) and purified with MicroSpin G-50 columns (Cytiva, Cat. # 27533001) using the manufacturers’ recommendations. The membrane was incubated with labelled probe and 250 µg/mL salmon sperm DNA (Roche, Cat. # 10223638103) at 65°C overnight in Perfecthyb Plus Hybridization Buffer (Millipore, Cat. # H7033-125ML), washed in 2xSSC plus 0.5% SDS, and exposed to X-ray film for 4 days.

### Embryonic stem cell targeting and mouse generation

C10 v6.5 ES cells were obtained from Rudolf Jaenisch. BPP ES cells were generated in our laboratory [32]. ES cells were electroporated with 15 µg targeting vector and 7.5 µg pCAGGS-FLPe and selected with 120 µg/mL Hygromycin (Invivogen, Cat # ant-hg-1). Clones were picked after 7–10 days, expanded, and verified by PCR genotyping of the targeted allele. The primers used for genotyping were PGK-forward 5’-GAGCAGCTGAAGCTTATGGA-3’ and Hygro-reverse 5’-CTGAATTCCCCAATGTCAAG-3’. A genomic region in the *Nras* gene was used as internal control (Nras-forward 5’-AGACGCGGAGACTTGGCGAGC-3’ and Nras-reverse 5’-GCTGGATCGTCAAGGCGCTTTTCC-3’). Targeted ES cells were injected into Balb/c blastocysts and transferred into pseudopregnant CD1 females. ES cell targeting and blastocyst injections were performed by the Moffitt Gene Targeting Core.

### Mouse husbandry and hydrodynamic tail vein injection

All animal experiments were conducted in accordance with an IACUC protocol (R-IS00005420) approved by the University of South Florida. For hydrodynamic tail vein injection, C57Bl/6 mice (Jackson Laboratory) were injected with a volume of sterile 0.9% saline solution (Fisher Scientific, Cat. # Z1376) equivalent to 10% of their body weight. The saline solution contained 10 µg spGFP transposon plasmid, 2.5 µg CMV-SB13, 10 µg pT3-EF1a-Myc-IRES-Luc, 10 µg sgp53-px330.

### Mouse melanoma cell line generation

Melanoma cells were isolated from two tumours from a TRE-spGFP BPP chimera, expanded *in vitro* for one week, and transplanted subcutaneously into athymic nude mice (The Jackson Laboratory, J:NU Stock #007850), followed by the establishment of cell lines from the secondary tumours. Melanoma cell isolation was performed as previously described [32]. The CAGS-LSL-rtTA3 allele was unrecombined in the established cell lines and to induce rtTA3 expression, cell lines were transduced with Ad5CMVCre adenovirus purchased from the University of Iowa Viral Vector Core (https://vector-core.medicine.uiowa.edu/). To induce spGFP expression, cells were cultured in the presence of 0.5 µg/mL Doxycycline for 48 hours.

### Statistical analysis

Statistical analysis was performed using the unpaired two-tailed t-test. All in vitro experiments were performed with three biological and three technical replicates. Unless otherwise noted, the mean ± SEM of one representative experiment is shown. A p-value below 0.05 was considered statistically significant.

## Results

### Limited utility of lentiviruses for circRNA overexpression

**Figure 1.**
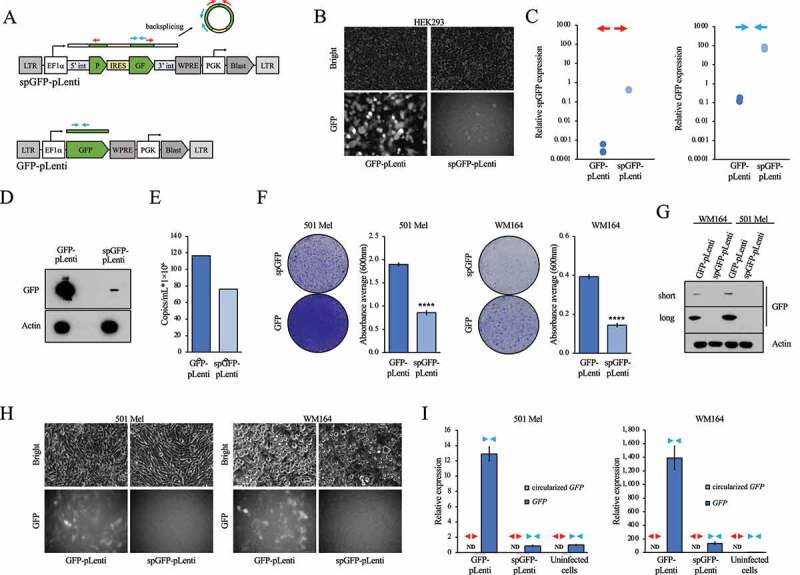
Lentiviral delivery of a circRNA expression construct. (A) Schematic outline of the circular spGFP (spGFP-pLenti) and linear GFP (GFP-pLenti) expression constructs. Red arrows indicate divergent, spGFP-specific primers, while blue arrows indicate convergent primers that detect both backspliced spGFP and linear GFP. LTR, long-terminal repeat; 5ʹint, human ZKSCAN1 5’ intron; 3ʹint, human ZKSCAN1 3’ intron; IRES, internal ribosome entry site; pA, polyadenylation signal; Blast, Blasticidin resistance. (B) Brightfield and green fluorescence images of virus-producing HEK293 cells transfected with either GFP-pLenti or spGFP-pLenti. Faint GFP positivity is evident in cells transfected with spGFP-pLenti, indicating backsplicing. (C) RT-qPCR showing the expression of circular spGFP (left panel) and linear/circular GFP (right panel) in virus-producing HEK293 cells transfected with either GFP-pLenti or spGFP-pLenti. Each dot represents one biological replicate. The divergent (red arrows) and convergent (blue arrows) primers from (A) that were used for these RT-qPCRs are indicated. (D) Western blot showing the expression of GFP in virus-producing HEK293 cells transfected with either GFP-pLenti or spGFP-pLenti. (E) Viral titre produced by HEK293 cells transfected with either GFP-pLenti or spGFP-pLenti measured by RT-qPCR. (F) Transduction of 501Mel and WM164 human melanoma cell lines with GFP-pLenti or spGFP-pLenti viral supernatants. Transduced cells were selected with 10 µg/mL Blasticidin for 1 week and then stained with Crystal Violet (left panels). The quantification of the extracted dye is shown in the right panels. (G) Western blot showing GFP expression in Blasticidin-selected WM164 and 501Mel cells transduced with GFP-pLenti or spGFP-pLenti. (H) Brightfield and green fluorescence images of Blasticidin-selected 501Mel and WM164 cells transduced with GFP-pLenti or spGFP-pLenti viral supernatants. (I) RT-qPCR showing the expression of linear/circular GFP and circular spGFP in 501Mel and WM164 cells transduced with GFP-pLenti or spGFP-pLenti viral supernatants. The divergent (red triangles) and convergent (blue triangles) primers used for the RT-qPCRs are indicated. Expression of spGFP was not detected (ND) with divergent primers. Untransduced parental cell lines are included as GFP-negative controls. ****, p < 0.0001.

To identify efficient approaches that enable long-term, stable expression of ectopic circRNAs, we first tested the utility of lentiviruses. To this end, we cloned a split-GFP (spGFP) reporter into the pLenti-Blasticidin lentiviral vector under the control of the CMV promoter (spGFP-pLenti). The spGFP reporter [30] contains a split enhanced GFP cDNA and an internal ribosome entry site (IRES) which allows for the production of GFP protein only when the mRNA is backspliced and a circular *GFP* RNA is generated. Circularization is mediated by the introns flanking the human ZKSCAN1 circRNA (Fig. 1A). We transfected the spGFP-pLenti vector along with VSVG envelope and ∆8.2 packaging plasmids into HEK293T cells for lentivirus production. We also transfected a pLenti-Blasticidin version carrying a regular GFP cDNA whose mRNA is not circularized (GFP-pLenti, Fig. 1A) into HEK293T cells for side-by-side comparison of lentiviral vectors carrying constructs that are backspliced (spGFP) or not spliced (GFP). GFP-pLenti-transfected HEK293T cells expressed GFP protein (Fig. 1B), which is expected due to translation of the viral transcript. Notably, HEK293T cells transfected with spGFP-pLenti also were GFP-positive, albeit at lower levels (Fig. 1B). In addition, circular *GFP* RNA was detected in spGFP-pLenti-transfected HEK293T cells by RT-qPCR using divergent primers that specifically detect backspliced *GFP* RNA (Fig. 1C). Circular *GFP* was not detected in GFP-pLenti-transfected HEK293T cells, while using convergent primers that do not distinguish between circular and linear transcripts detected *GFP* RNA in cells transfected with spGFP-pLenti or GFP-pLenti (Fig. 1C). We also detected GFP protein in spGFP-pLenti-transfected HEK293T cells by Western blot (Fig. 1D). These results indicate that the spGFP-pLenti viral transcript is backspliced prior to packaging into viral capsids, which may interfere with efficient virus production. Alternatively, a spliced viral RNA lacking the circRNA could be packaged into viral capsids. This spliced viral RNA would consist of the circRNA-flanking sequences connected via the branch point in the ZKSCAN1 5ʹintron and therefore would be ‘kinked’ and likely dysfunctional. To test this, we first estimated the viral titre by measuring viral RNA by standard curve RT-qPCR in the viral supernatant. The viral titre produced by spGFP-pLenti was comparable to that produced by GFP-pLenti (Fig. 1E), indicating that backsplicing does not impair the generation of particles containing viral RNA per se. We then transduced two human melanoma cell lines, WM164 and 501Mel, with the spGFP-pLenti and GFP-pLenti viral particles and selected the cells in 10 µg/mL Blasticidin for 10 days. Notably, despite the comparable viral titres, melanoma cells transduced with the spGFP-pLenti virus preparation died significantly more during Blasticidin selection than cells transduced with GFP-pLenti (Fig. 1F). WM164 and 501Mel cells transduced with spGFP-pLenti that survived Blasticidin selection were negative for GFP by Western blot (Fig. 1G) and fluorescence imaging (Fig. 1H). Moreover, we could not detect circularized *spGFP* by RT-qPCR using divergent primers and only very minor amounts of *GFP* using convergent primers that detect circularized and non-circularized *GFP* (Fig. 1I). Overall, these findings suggest that lentiviruses are not conducive to the generation of stable cell lines overexpressing circRNAs because of the undesired splicing of viral mRNA in virus-producing cells.

### Stable circRNA overexpression using a transposon

**Figure 2.**
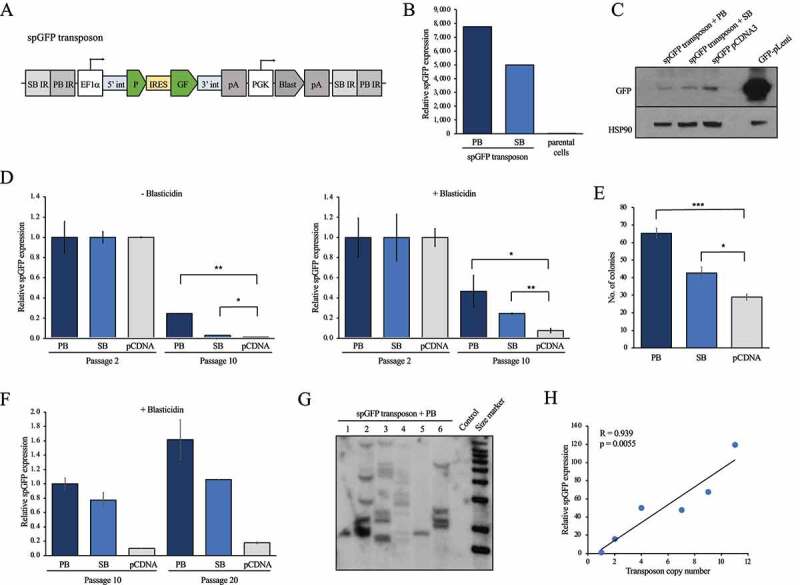
circRNA construct delivery and stable expression with a transposon. (A) Schematic outline of the spGFP transposon. SB IR, inverted repeat for Sleeping Beauty transposase; PB IR, inverted repeat for piggyBac transposase; 5ʹint, human ZKSCAN1 5’ intron; 3ʹint, human ZKSCAN1 3’ intron; IRES, internal ribosome entry site; pA, polyadenylation signal; Blast, Blasticidin resistance. (B) RT-qPCR showing expression of circular *spGFP* in HEK293 cells transfected with the spGFP transposon and either piggyBac (PB) or Sleeping Beauty (SB) expression vectors. Untransfected parental cells are included as negative controls. (C) Western blot showing expression of GFP in HEK293 cells transfected with the spGFP transposon and either piggyBac (PB) or Sleeping Beauty (SB) expression vectors. A circular spGFP expression plasmid (spGFP-pCDNA3) and a linear GFP (GFP-pLenti) lentiviral vector were transfected into HEK293 and used as controls for GFP expression. (D) RT-qPCR showing circular *spGFP* expression in HEK293 cells transfected with the spGFP transposon and either piggyBac (PB) or Sleeping Beauty (SB) or empty pCDNA3. Circular *spGFP* expression was analysed 2 and 10 passages after transfection in either unselected cells (left panel) or cells kept on 10 µg/mL Blasticidin (right panel). (E) Quantification of Crystal Violet-stained colonies of human A375 melanoma cells transfected with the spGFP transposon and either piggyBac (PB) or Sleeping Beauty (SB) or empty pCDNA3. Cells were cultured for 10 passages and then selected in 10 µg/mL Blasticidin. (F) RT-qPCR showing expression of circular *spGFP* in A375 cells transfected with the spGFP transposon and either piggyBac (PB) or Sleeping Beauty (SB) or empty pCDNA3. Cells were cultured for 10 passages, followed by another 10 passages in the presence of 10 µg/mL Blasticidin (Passage 20). (G) Southern blot demonstrating the integration of the spGFP transposon in A375 clones transfected with spGFP transposon vector and a piggyBac expression plasmid. Untransfected parental cells were used as control. (H) Correlation of *spGFP* expression as measured by RT-qPCR and transposon copy number in the A375 clones shown in (G). *, p < 0.05; **, p < 0.01; ***, p < 0.001.

Backsplicing of the viral transcript in virus-producing HEK293 cells may interfere with the production of viral particles carrying an intact, non-spliced circRNA transgene. We therefore decided to test a non-viral circRNA transgene delivery method using a transposon. We modified the ATP1 transposon [33] harbouring inverted repeats for the Sleeping Beauty and piggyBac transposases to contain the spGFP reporter under the control of the EF1α promoter and a Blasticidin selection cassette (Fig. 2A). We transfected HEK293 cells with the spGFP transposon along with expression plasmids encoding the Sleeping Beauty or piggyBac transposases. Both combinations resulted in robust expression of circularized *spGFP*, as determined by RT-qPCR with divergent primers (Fig. 2B). In addition, we observed expression of GFP by Western blot in these cells (Fig. 2C). Next, we tested whether circular *spGFP* expression is maintained over multiple passages, which would indicate that copies of the spGFP transposon are integrated into the genome. To this end, we transfected HEK293 cells with the spGFP transposon and either Sleeping Beauty or piggyBac expression vectors or empty pCDNA3 and determined the expression of circular *spGFP* at 2 and 10 passages post-transfection. Both in the presence and absence of Blasticidin, circular *spGFP* expression declined after 10 passages (Fig. 2D). However, in the presence of Sleeping Beauty and especially piggyBac, *spGFP* levels were maintained at higher levels compared to the empty pCDNA3-transfected control (Fig. 2D). We next tested whether a similar effect is observed in human melanoma cells by transfecting A375 cells with the spGFP transposon and either Sleeping Beauty or piggyBac expression vectors or empty pCDNA3. The transfected A375 cells were cultured for 10 passages in the absence of Blasticidin and then placed on Blasticidin selection. Significantly more cells survived selection when Sleeping Beauty or piggyBac were expressed (Fig. 2E), suggesting the transposon was maintained long-term in the absence of selection. Accordingly, the presence of piggyBac or Sleeping Beauty mediated increased expression of circular *spGFP* in Blasticidin-selected A375 cells (Fig. 2F). Interestingly, *spGFP* expression levels were stable when these A375 cells were maintained for another 10 passages in the presence of Blasticidin (Fig. 2F), demonstrating that long-term circRNA expression is stable.

**Figure 3.**
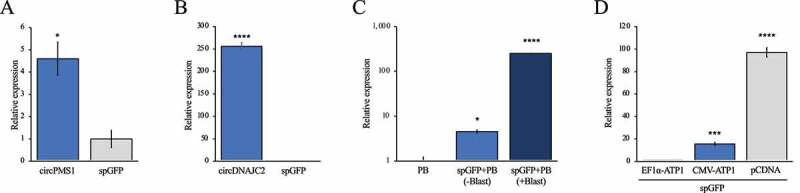
Delivery of human circRNAs using the transposon approach. (A) Expression of *circPMS1* in WM164 melanoma cells transfected with the circPMS1 transposon and a piggyBac expression plasmid. (B) Expression of *circDNAJC2* in WM164 melanoma cells transfected with the circDNAJC2 transposon and a piggyBac expression plasmid. (C) Expression of circular *spGFP* in NIH3T3 fibroblasts transfected with piggyBac (PB) alone or piggyBac and the spGFP transposon (PB+spGFP). PB+spGFP cells were analysed before (-Blast) and after (+Blast) Blasticidin selection. (D) RT-qPCR showing the expression of *spGFP* in HEK293 cells transfected with the EF1α-spGFP or CMV-spGFP transposons and piggyBac or with spGFP-pCDNA3. *, p < 0.05; ****, p < 0.0001.

To test whether transposon maintenance was due to genomic integration, we isolated six Blasticidin-selected single cell A375 clones that were transfected with the spGFP transposon and piggyBac expression plasmid and isolated genomic DNA. We digested the DNA with MspI to cut within the transposon and in juxtaposed genomic regions and subjected the DNA to Southern blot analysis using a Blasticidin probe. This strategy detected multiple bands of different sizes in most clones (Fig. 2G), validating that the transposon was integrated in the genome. We also assessed the levels of circular *spGFP* in these six A375 clones and observed that circular *spGFP* levels strongly correlated with the number of integrated transposons (Fig. 2H). To confirm that the transposon is amenable for delivering other circRNA transgenes, we replaced the spGFP construct with the exons of the human circDNAJC2 and circPMS1 circular RNAs. We delivered these circRNA transposon constructs along with piggyBac to WM164 cells and selected in Blasticidin. This resulted in significant overexpression of *circDNAJC2* and *circPMS1* as determined by RT-qPCR using divergent primers (Fig. 3A,B). To test if circRNAs can be delivered also to murine cells using the transposon approach, we co-transfected NIH3T3 fibroblasts with piggyBac and the spGFP transposon. Circular *spGFP* expression was detected in NIH3T3 cells co-transfected with piggyBac and the spGFP transposon compared to cells transfected with piggyBac alone, and the *spGFP* expression was further increased upon selection with Blasticidin (Fig. 3C). Finally, we compared *spGFP* expressed achieved with the transposon in HEK293 cells to *spGFP* expression from the pCDNA3 plasmid used for transient transfection. While *spGFP* expression was significantly lower from the transposon, replacing the EF1α promoter with a CMV promoter increased *spGFP* expression approximately 15-fold (Fig. 3D). Taken together, stable circRNA overexpression is readily achieved in human and murine cells using a transposon as delivery vehicle.

### In vivo delivery of a circRNA construct using transposons

**Figure 4.**
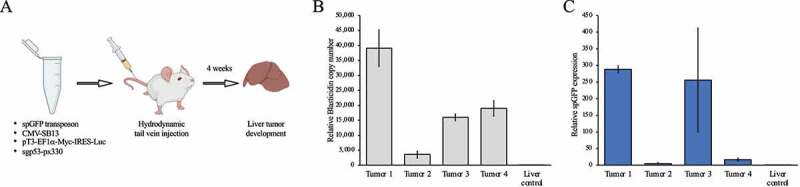
Ectopic circRNA expression in a hepatocellular carcinoma model with transposon-based delivery. (A) Outline of the hydrodynamic tail vein injection to generate a hepatocellular carcinoma model. (B) qPCR copy number analysis on genomic DNA isolated from liver tumours to detect Blasticidin as a surrogate for the presence of the spGFP transposon. Normal liver from uninjected mice was used as negative control. (C) RT-qPCR analysis on RNA isolated from liver tumours using divergent primers to quantify the expression of circular *spGFP*. Normal liver from uninjected mice was used as negative control.

Following the establishment of a circRNA transposon expression system in cultured cells, we assessed whether the transposon approach is suitable for delivering circRNA overexpression constructs *in vivo*. Specifically, we examined whether a circRNA construct can be delivered to the liver of mice via hydrodynamic tail vein injection. To enrich for cells that contain the circRNA construct, we used a hepatocellular carcinoma model in which tumorigenesis is driven by delivering a Sleeping Beauty transposon harbouring a c-Myc cDNA [34]. We combined the spGFP transposon plasmid with a Sleeping Beauty transposase expression plasmid, the c-Myc transposon, and a plasmid encoding Cas9 and a sgRNA targeting p53 [34] and performed hydrodynamic tail vein injections in wildtype C57BL/6 mice (Fig. 4A). After ageing the mice for 4 weeks we isolated the livers and out of the 5 mice injected, 2 mice had developed hepatocellular carcinomas. We first isolated genomic DNA from 4 liver tumours and performed qPCRs using a primer pair designed over the Blasticidin cassette. This demonstrated that the spGFP transposon DNA was present in the tumours (Fig. 4B). Next, we isolated RNA from the same liver tumours and performed a RT-qPCR for circular *spGFP* using divergent primers. All tumours tested exhibited expression of circular *spGFP*, albeit at varying levels (Fig. 4C). These data demonstrate that circRNAs can be ectopically expressed in mouse liver by delivering circRNA transgene-containing transposons via hydrodynamic tail vein injection.

### Global circRNA overexpression in genetically engineered mice

**Figure 5.**
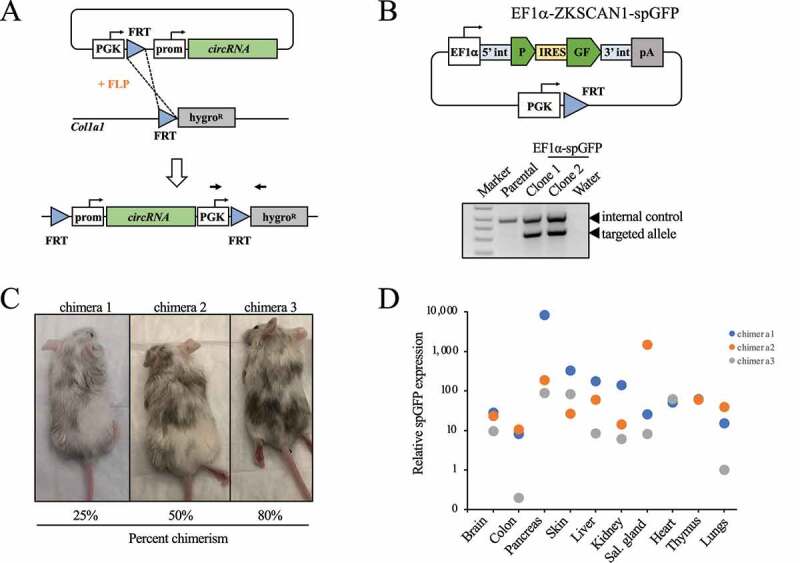
Global ectopic circRNA expression in genetically engineered knock-in mice. (A) Schematic outline of the recombination-mediated cassette exchange (RMCE) approach. Genotyping primers in the PGK promoter (forward) and Hygromycin (reverse) are indicated. (B) Top: schematic outline of the constitutive and ubiquitous circular spGFP targeting vector (EF1α-ZKSCAN1-spGFP). 5ʹint, human ZKSCAN1 5’ intron; 3ʹint, human ZKSCAN1 3’ intron; IRES, internal ribosome entry site; pA, polyadenylation signal. Bottom: genotyping PCR to confirm successful targeting of ES cells by RMCE. PGK-Hygromycin primers indicated in (A) were used to detect the targeted allele. (C) Images of the three EF1α-ZKSCAN1-spGFP chimeras. The estimated percentage of chimerism based on coat colour is indicated. (D) RT-qPCR showing circular spGFP expression in organs from EF1α-ZKSCAN1-spGFP chimeras. Sal. gland, salivary gland.

While the *in vivo* delivery of transposon DNA is readily achievable in livers, it is far more difficult to accomplish in other organs and cell types. To study gene functions *in vivo*, overexpression constructs are typically inserted into the mouse germline of genetically engineered mice. However, to our knowledge this approach has not been used for *in vivo* overexpression of circRNAs. Thus, we tested whether an ectopic circRNA knock-in allele is functional in mice. To this end, we cloned a targeting vector for recombination-mediated cassette exchange (RMCE) into embryonic stem cells (ESCs) harbouring a homing cassette (CHC) downstream of the collagen1a1 locus (Fig. 5A). This targeting vector contains the spGFP circRNA reporter under the control of the constitutive EF1α promoter as well as a FRT site and a PGK promoter to render it compatible with RMCE (Fig. 5B). We targeted C10 v6.5 ESCs [35] with this spGFP expression construct and validated correct transgene integration by PCR (Fig. 5B). We then injected one positively targeted ESC clone into Balb/c blastocysts followed by transfer into pseudopregnant CD1 females. Three chimeras were born with ESC contribution based on coat-colour ranging from 25–80% (Fig. 5C). We euthanized the chimeras at 4 weeks of age and collected all major organs, isolated RNA, and performed RT-qPCR to detect *spGFP* expression with divergent primers. Notably, *spGFP* expression was detected in all organs (Fig. 5D). Thus, using a ubiquitous promoter and the human ZKSCAN1 introns enables expression of circRNAs in various organs of knock-in mice.

### Tissue-specific and inducible circRNA expression in a mouse melanoma model

Having achieved global expression of the spGFP reporter circRNA in mice, we assessed whether ectopic circRNA expression can be induced and directed to a specific cell type *in vivo*. To this end, we used our ESC-genetically engineered mouse modelling (ESC-GEMM) melanoma platform [32] consisting of ESCs derived from GEMMs harbouring alleles to efficiently induce melanoma formation, integrate transgenes by RMCE, and control transgene expression with the Tet-ON system. Specifically, we used the BPP model, which harbours LSL-Braf^V600E^ and Pten^FL/FL^ driver alleles, a melanocyte-specific, 4OH-Tamoxifen (4OHT)-inducible Tyr-CreERt2 recombinase allele, the Cre-dependent CAGs-LSL-rtTA3 transactivator, and the CHC homing allele. When chimeric mice generated with the BPP model are topically treated with 4OHT on their back skin, melanomas form within 6 weeks. Moreover, expression of inducible transgenes is activated specifically in melanoma cells by switching chimeras to a Dox-containing diet. This model is thus ideal to rapidly test if ectopic circRNA expression can be directed to melanoma cells.

**Figure 6.**
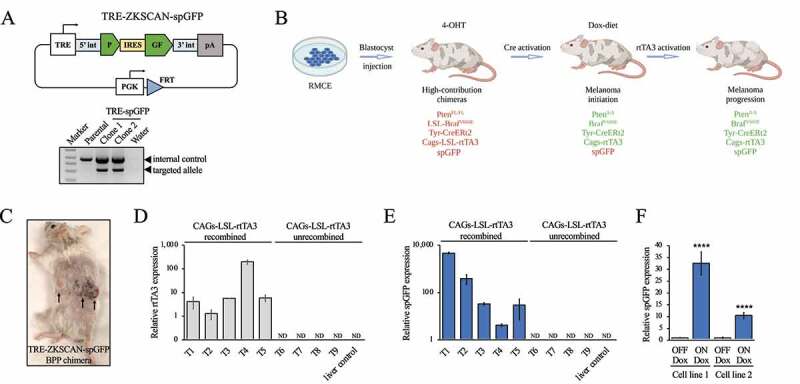
Melanoma cell-specific ectopic circRNA expression in a genetically engineered melanoma mouse model. (A) Top: schematic outline of the Dox-inducible circular spGFP targeting vector (TRE-ZKSCAN1-spGFP). TRE, Tetracycline-response element promoter; 5ʹint, human ZKSCAN1 5’ intron; 3ʹint, human ZKSCAN1 3’ intron; IRES, internal ribosome entry site; pA, polyadenylation signal. Bottom: genotyping PCR to confirm successful targeting of ES cells by RMCE. PGK-Hygromycin primers indicated in Fig. 5A were used to detect the targeted allele. (B) Outline of the ESC-GEMM approach. BPP ES cells are targeted with the circRNA expression construct by RMCE. Targeted ES cells are used to generate chimeras, which are treated with 4-OH Tamoxifen (4-OHT) to activate melanocyte-specific Cre recombinase. Cre induces expression of Braf^V600E^ and rtTA3 and deletes Pten. Subsequent administration of Doxycycline activates the Tet-ON system and induces expression of the circRNA expression construct. (C) Image of a BPP chimera harbouring the TRE-ZKSCAN1-spGFP construct. Melanomas are indicated by arrows. (D) RT-qPCR showing the expression of rtTA3 in melanomas. Tumours in which the CAGs-LSL-rtTA3 allele is recombined express the rtTA3 transactivator. (E) RT-qPCR showing the expression of circular *spGFP* in melanomas. Melanomas that express rtTA3 also express *spGFP*. (F) Expression of *spGFP* was analysed by RT-qPCR in TRE-spGFP BPP melanoma cell lines in the absence or presence of 0.5 µg/mL Doxycycline. ND, not detected; ****, p < 0.0001.

To generate an inducible spGFP circRNA reporter targeting vector, we replaced the EF1α promoter with a Doxycycline-inducible TRE promoter (Fig. 6A). We targeted BPP ESCs with the TRE-spGFP construct by RMCE (Fig. 6A) and produced chimeric mice via blastocyst injection (Fig. 6B). At weaning, we topically applied to the shaved back skin either several 1 µL drops of 25 mg/mL 4OHT in DMSO or enough 4OHT solution (2.5 mg/mL) to cover the back skin. After 5–6 weeks when melanomas had formed, we switched chimeras from a regular diet to a 200 mg/kg Dox diet for one week. We then harvested melanomas (Fig. 6C) and isolated RNA. As there is no selective pressure to recombine the CAGs-LSL-rtTA3 allele in contexts where the inserted transgene does not accelerate melanomagenesis [32], we first validated CAGs-LSL-rtTA3 recombination. To do so, we assessed expression of rtTA3 in melanomas by RT-qPCR. Of the 9 isolated tumours, 5 had the CAGs-LSL-rtTA3 allele recombined and expressed rtTA3, while 4 tumours did not (Fig. 6D). We then determined if circular *spGFP* was expressed in melanomas by RT-qPCR using divergent primers. Notably, circular *spGFP* was detected in Dox-treated tumours that also express rtTA3 (Fig. 6E) while circular *spGFP* expression was not detected in Dox-treated tumours that lacked rtTA3 expression or in livers (Fig. 6E). To further test the inducibility of circRNA expression, we established two melanoma cell lines from TRE-spGFP chimeras. When cultured in the presence of 0.5 µg/mL Dox, circular *spGFP* expression was induced in both cell lines (Fig. 6F). Thus, ectopic circRNA expression can be spatiotemporally controlled *in vivo* by using the appropriate Cre strain and the Tet-ON system.

## Discussion

In this study we established new genetic tools for the stable, long-term overexpression of candidate circRNAs in cultured mammalian cells *in vitro* and in mice. We used a transposon approach to deliver a circRNA expression cassette to cell lines, where it is integrated into the genome upon transient co-expression of a transposase. We further show that circRNA expression cassettes can be delivered to the livers of mice via hydrodynamic tail vein injections. In addition, using a recombination-mediated cassette exchange approach, we inserted a circRNA expression cassette in the genome of mouse ESCs, enabling the generation of mice that ubiquitously express an ectopic circRNA. We used the same approach to generate a melanoma mouse model in which ectopic circRNA expression can be induced specifically in melanoma cells. These tools and approaches will pave the way for functional analyses of circRNA function and overcome some of the limitations of current circRNA expression systems. In particular, we are unaware of any attempts to overexpress circRNAs *in vivo* and our work demonstrates the feasibility of generating circRNA transgenic mice. This will stimulate further research of circRNA function in normal physiology and disease.

We observed that circRNA expression cassettes in lentiviral constructs are backspliced in the virus-producing cells. This may interfere with packaging the viral transcript into viral particles. Moreover, viral transcripts that have undergone backsplicing and are packaged into viral particles would lack the circRNA expression cassette. These considerations offer possible explanations as to why we observed a high viral titre with the spGFP-pLenti lentiviral construct but failed to obtain appreciable Blasticidin resistance or circular *spGFP* expression in melanoma cells transduced with the viral supernatant. The extent of backsplicing in virus-producing HEK293cells is likely dictated, at least in part, by the introns used to mediate circularization. The human ZKSCAN1 introns used in our construct efficiently promote circularization [30], which in this case is a detriment. The ZKSCAN1 introns could be replaced by introns that less efficiently mediate circularization to prevent backsplicing in HEK293 cells. However, this would also reduce the extent of circularization in transduced cells and increase the abundance of unspliced linear viral transcript containing the circRNA exons and flanking introns. It may therefore be challenging to discern whether any observed phenotype is elicited by the circRNA or the linear transcript. One could use RNAi or inhibitors targeting the splicing machinery to transiently reduce splicing in virus-producing cells. However, this may negatively affect HEK293 cells and thereby lower the quality of the produced virus. We found the transposon approach to be an easy-to-use alternative to lentiviruses for stable circRNA overexpression.

Given that the transposon is not delivered to target cells via an RNA intermediate, this approach is not hindered by the shortcomings of lentiviral circRNA delivery. However, as the transposon and transposase expression plasmids are co-transfected, this approach may be limited in difficult-to-transfect cell types. Nevertheless, we observed long-term expression of the circular spGFP reporter in melanoma cell lines using two different transposases, Sleeping Beauty and piggyBac. Interestingly, we observed that circular *spGFP* expression decreased in the first 10 passages post-transfection. This is likely explained by the presence of donor plasmids that have retained the transposon. Such plasmids contribute to *spGFP* expression but are lost from the cells over time. The modular nature of the transposon allows for the easy exchange of the promoter, the circularizing introns, or the selection marker, allowing for the generation of custom-built transposons that fit one’s need. For instance, we showed that replacing the EF1α promoter with a CMV promoter increased *spGFP* expression 15-fold. One may also wish to optimize the DNA delivery and transposon:transposase plasmid ratios to control for the number of integrated transposons per cell in a polyclonal cell population. As the transposon copy number correlates with the extent of ectopic circRNA expression, normalizing integration events would lead to more homogeneous expression levels. As an alternative, individual single cell clones may be selected that exhibit the desired transposon copy number and/or circRNA expression levels. Finally, to ensure that any observed phenotypes are not due to insertional mutagenesis but rather expression of the circRNA of interest, strategies to activate or inactivate ectopic circRNA expression using Tet-ON/OFF or Cre/loxP approaches could be used in combination with transposon-mediated delivery. Thus, the approaches and controls for generating stable cell lines with lenti- or retroviruses must also be applied when using transposons as a delivery method.

We successfully generated genetically engineered mice in which an ectopic circRNA was expressed constitutively in all cells or inducibly in melanocytes/melanoma cells. Given that ectopic circRNA expression was possible with both approaches, we surmise that most, if not all, gene targeting approaches to produce overexpression mouse strains are compatible with circRNA expression constructs. circRNA depletion via genetic knock-out, as has been done for CDR1as [36], is feasible and useful for transcripts that are primarily circularized like CDR1as [3,37]. However, most circRNAs are encoded by genes that also encode protein-coding mRNAs. The knock-out of circRNA exons in those genes will therefore also affect the encoded protein, which complicates the analysis of the observed phenotypes. Sophisticated methods that target the flanking introns to prevent circularization without affecting linear splicing could be developed. For instance, one could employ a CRISPR approach to mutate or delete AluI sites or other repeat sequences that mediate circularization. Similarly, binding sites of RNA-binding proteins that facilitate backsplicing could be mutated using the same approach. The efficiency of these approaches remains to be tested and is likely going to be locus dependent. Thus, circRNA overexpression in genetically engineered mice to study circRNA function *in vivo* is currently the more practical approach. In summary, the tools and approaches developed in this study expand the genetic toolkit to overexpress circRNAs *in vitro* and *in vivo* and will stimulate studies to functionally characterize circRNAs.

## Data Availability

Data sharing is not applicable to this article as no new data were created or analyzed in this study. Plasmids will be deposited at Addgene. Further information and requests for resources and reagents should be directed to and will be fulfilled by the lead contact, Florian A. Karreth (florian.karreth@moffitt.org).

## References

[1] Guo JU, Agarwal V, Guo H, et al. Expanded identification and characterization of mammalian circular RNAs. Genome Biol. 2014;15(7):409. 25070500 10.1186/s13059-014-0409-z PMC4165365

[2] Jeck WR, Sorrentino JA, Wang K, et al. Circular RNAs are abundant, conserved, and associated with ALU repeats. RNA. 2013;19(2):141–157. 23249747 10.1261/rna.035667.112 PMC3543092

[3] Memczak S, Jens M, Elefsinioti A, et al. Circular RNAs are a large class of animal RNAs with regulatory potency. Nature. 2013;495(7441):333–338. 23446348 10.1038/nature11928

[4] Rybak-Wolf A, Stottmeister C, Glažar P, et al. Circular RNAs in the Mammalian Brain Are Highly Abundant, Conserved, and Dynamically Expressed. Mol Cell. 2015;58(5):870–885. 25921068 10.1016/j.molcel.2015.03.027

[5] Venø MT, Hansen TB, Venø ST, et al. Spatio-temporal regulation of circular RNA expression during porcine embryonic brain development. Genome Biol. 2015;16(1):245. 26541409 10.1186/s13059-015-0801-3 PMC4635978

[6] Salzman J, Chen RE, Olsen MN, et al. Cell-type specific features of circular RNA expression. Moran JV, editor. PLoS Genet. 2013;9(9):e1003777. 24039610 10.1371/journal.pgen.1003777 PMC3764148

[7] Salzman J, Gawad C, Wang PL, et al. Circular RNAs are the predominant transcript isoform from hundreds of human genes in diverse cell types. Preiss T, editor. PloS one. 2012;7(2):e30733. 22319583 10.1371/journal.pone.0030733 PMC3270023

[8] Szabo L, Morey R, Palpant NJ, et al. Statistically based splicing detection reveals neural enrichment and tissue-specific induction of circular RNA during human fetal development. Genome Biol. 2015;16(1):126. 26076956 10.1186/s13059-015-0690-5 PMC4506483

[9] You X, Vlatkovic I, Babic A, et al. Neural circular RNAs are derived from synaptic genes and regulated by development and plasticity. Nat Neurosci. 2015;18(4):603–610. 25714049 10.1038/nn.3975 PMC4376664

[10] Westholm JO, Miura P, Olson S, et al. Genome-wide analysis of drosophila circular RNAs reveals their structural and sequence properties and age-dependent neural accumulation. Cell Rep. 2014;9(5):1966–1980. 25544350 10.1016/j.celrep.2014.10.062 PMC4279448

[11] Hansen TB, Jensen TI, Clausen BH, et al. Natural RNA circles function as efficient microRNA sponges. Nature. 2013;495(7441):384–388. 23446346 10.1038/nature11993

[12] Du WW, Fang L, Yang W, et al. Induction of tumor apoptosis through a circular RNA enhancing Foxo3 activity. Cell Death Differ. 2017;24(2):357–370. 27886165 10.1038/cdd.2016.133 PMC5299715

[13] Holdt LM, Stahringer A, Sass K, et al. Circular non-coding RNA ANRIL modulates ribosomal RNA maturation and atherosclerosis in humans. Nat Commun. 2016;7(1):12429. 27539542 10.1038/ncomms12429 PMC4992165

[14] Abdelmohsen K, Panda AC, Munk R, et al. Identification of HuR target circular RNAs uncovers suppression of PABPN1 translation by CircPABPN1. RNA Biology. 2017;14(3):361–369. 28080204 10.1080/15476286.2017.1279788 PMC5367248

[15] Yang Q, Du WW, Wu N, et al. A circular RNA promotes tumorigenesis by inducing c-myc nuclear translocation. Cell Death Differ. 2017;24(9):1609–1620. 28622299 10.1038/cdd.2017.86 PMC5563992

[16] Yang Z-G, Awan FM, Du WW, et al. The circular RNA interacts with STAT3, increasing its nuclear translocation and wound repair by modulating Dnmt3a and miR-17 function. Mol ther. 2017;25(9):2062–2074. 28676341 10.1016/j.ymthe.2017.05.022 PMC5589065

[17] Conn VM, Hugouvieux V, Nayak A, et al. A circRNA from SEPALLATA3 regulates splicing of its cognate mRNA through R-loop formation. Nat Plants. 2017;3(5):17053. 28418376 10.1038/nplants.2017.53

[18] Zhang Y, Zhang X-O, Chen T, et al. Circular intronic long noncoding RNAs. Mol Cell. 2013;51(6):792–806. 24035497 10.1016/j.molcel.2013.08.017

[19] Ashwal-Fluss R, Meyer M, Pamudurti NR, et al. circRNA biogenesis competes with pre-mRNA splicing. Mol Cell. 2014;56(1):55–66. 25242144 10.1016/j.molcel.2014.08.019

[20] Li Z, Huang C, Bao C, et al. Exon-intron circular RNAs regulate transcription in the nucleus. Nat Struct Mol Biol. 2015;22(3):256–264. 25664725 10.1038/nsmb.2959

[21] Pamudurti NR, Bartok O, Jens M, et al. Translation of CircRNAs. Mol Cell. 2017;66(1):9–21.e7. 28344080 10.1016/j.molcel.2017.02.021 PMC5387669

[22] Zhang M, Huang N, Yang X, et al. A novel protein encoded by the circular form of the SHPRH gene suppresses glioma tumorigenesis. Oncogene. 2018;37(13):1805–1814. 29343848 10.1038/s41388-017-0019-9

[23] Zhang M, Zhao K, Xu X, et al. A peptide encoded by circular form of LINC-PINT suppresses oncogenic transcriptional elongation in glioblastoma. Nat Commun. 2018;9(1):4475. 30367041 10.1038/s41467-018-06862-2 PMC6203777

[24] Yang Y, Gao X, Zhang M, et al. Novel role of FBXW7 circular RNA in repressing glioma tumorigenesis. JNCI: Journal of the National Cancer Institute. 2018;110(3):304–315. 10.1093/jnci/djx166 PMC6019044 28903484

[25] Liang W-C, Wong C-W, Liang -P-P, et al. Translation of the circular RNA circβ-catenin promotes liver cancer cell growth through activation of the Wnt pathway. Genome Biol. 2019;20(1):84. 31027518 10.1186/s13059-019-1685-4 PMC6486691

[26] Zheng X, Chen L, Zhou Y, et al. A novel protein encoded by a circular RNA circPPP1R12A promotes tumor pathogenesis and metastasis of colon cancer via Hippo-YAP signaling. Mol Cancer. 2019;18(1):47. 30925892 10.1186/s12943-019-1010-6 PMC6440158

[27] Yang Y, Fan X, Mao M, et al. Extensive translation of circular RNAs driven by N6-methyladenosine. Cell Res. 2017;27(5):626–641. 28281539 10.1038/cr.2017.31 PMC5520850

[28] Konermann S, Lotfy P, Brideau NJ, et al. Transcriptome Engineering with RNA-Targeting Type VI-D CRISPR Effectors. Cell. 2018;173(3):665–676.e14. 29551272 10.1016/j.cell.2018.02.033 PMC5910255

[29] Zhang Y, Nguyen TM, Zhang X-O, et al. Optimized RNA-targeting CRISPR/Cas13d technology outperforms shRNA in identifying functional circRNAs. Genome Biol. 2021;22(1):41. 33478577 10.1186/s13059-021-02263-9 PMC7818937

[30] Kramer MC, Liang D, Tatomer DC, et al. Combinatorial control of Drosophila circular RNA expression by intronic repeats, hnRNPs, and SR proteins. Genes Dev. 2015;29(20):2168–2182. 26450910 10.1101/gad.270421.115 PMC4617980

[31] Liang D, Wilusz JE. Short intronic repeat sequences facilitate circular RNA production. Gene Dev. 2014;28(20):2233–2247. 25281217 10.1101/gad.251926.114 PMC4201285

[32] Bok I, Vera O, Xu X, et al. A versatile ES cell-based melanoma mouse modeling platform. Cancer Res. 2019;80:canres.2924.2019–921. 10.1158/0008-5472.CAN-19-2924 PMC7024666 31744817

[33] Rad R, Rad L, Wang W, et al. PiggyBac transposon mutagenesis: a tool for cancer gene discovery in Mice. Science (New York, NY). 2010;330(6007):1104–1107. 10.1126/science.1193004 PMC3719098 20947725

[34] de GMR, Bresnahan E, Molina-Sánchez P, et al. β-catenin activation promotes immune escape and resistance to Anti–PD-1 therapy in hepatocellular carcinoma. Cancer Discov. 2019;9(8):1124–1141. 31186238 10.1158/2159-8290.CD-19-0074 PMC6677618

[35] Beard C, Hochedlinger K, Plath K, et al. Efficient method to generate single-copy transgenic mice by site-specific integration in embryonic stem cells. Genesis (New York, N.Y.: 2000). 2006;44(1):23–28. 10.1002/gene.20180 16400644

[36] Piwecka M, Glažar P, Hernandez-Miranda LR, et al. Loss of a mammalian circular RNA locus causes miRNA deregulation and affects brain function. Science (New York, NY). 2017;357(6357):eaam8526. 10.1126/science.aam8526 28798046

[37] Hansen TB, Wiklund ED, Bramsen JB, et al. miRNA-dependent gene silencing involving Ago2-mediated cleavage of a circular antisense RNA. Embo J. 2011;30(21):4414–4422. 21964070 10.1038/emboj.2011.359 PMC3230379

